# Some Considerations on the Early Diagnosis and Treatment of Mammary Cancer

**Published:** 1912-09

**Authors:** Charles W. Bonney

**Affiliations:** Philadelphia; 1117 Spruce Street


### Some Considerations on the Early Diagnosis and Treatment of Mammary Cancer

By CHARLES W. BONNEY, A. B., M. D.
Philadelphia.

AMONG the multifarious duties devolving upon the overworked general practitioner of medicine it is doubtful if there be one which entails greater responsibility than the recognition of malignant disease in its incipient stage. Insidious in its onset, slow in its development, and frequently long unproductive of troublesome, not so say alarming symptoms, carcinoma pursues its fell course unremittingly, unrelentingly, and all too often disastrously, before the unfortunate victim of its ravages comes to realize that within his body there is harbored an invading host which must eventually prove destructive to all vital processes, unless perchance it be not so solidly intrenched as to defy and render impotent every effort to dislodge it and rout it even unto the remotest vestige.

That the site which this, our most dreaded foe, chooses to select for its base of operations naturally influences its resistance to the onslaught of the surgeon, is well known to all who have had to deal with it in its various localizations. Thus it is that while the discovery of an internal cancer oftimes immediately banishes hope from our minds, the detection of a similar external neoplasm may leave us not without some expectation of good for the patient's future.

Of all forms of carcinoma except the superficial epithelioma, and the primary laryngeal form, the mammary is probably the most amenable to radical surgery, and it is for this reason that I have decided, in response to your invitation, to call to your attention certain considerations concerning its early diagnosis and modern treatment. So far as improvements in methods of operating are concerned, little if anything is to be expected, for the reason that the most approved methods now in vogue fulfill all requirements to a very high degree. In fact I believe that the operation I shall presently outline represents the acme of technical perfection. It is rather from increased diagnostic skill on the part of the family doctor that the surgeon will hope to obtain better results.

---

*Read before the McKean County Medical Society at Bradford, Pa., June 10, 1912

A large percentage of women affected with mammary cancer first consult the doctor because they have accidentlly discovered that there is a lump in one breast, and as it often has produced no annoyance they may have deferred their visit for some time after they first became aware of its presence. It is exactly in such early cases as these, gentlemen, cases which almost every doctor sees occasionally, just as he now and then sees cases of locomotor ataxia, diabetic gangrene and osteomyelitis for instance, that much can be done by prompt recognition of the nature of the tissue changes and sound advice as to the best method of dealing with them.

In these early cases how is malignancy to be recognized? In the first place there is one most important point upon which too much stress cannot be placed, namely, that at least eighty per cent. and probably more of all mammary tumors are cancers. This percentage was the one which obtained in five thousand cases of neoplasms of the breast which I had occasion to collect and analyze for Dr. Rodman a few years ago. More recent statistics show even a higher percentage, and it is not improbable that eighty-five out of every hundred are cancers. This is truly a point which I cannot too forcibly impress upon you. Such being the case, would it not be more sensible to assume that a suspicious swelling, particularly in a woman past forty-five, is malignant and give her the benefit of a skilled opinion at once rather than to advise her to wait and see what will happen? Fortunately the signs of malignancy are sufficiently well pronounced in the majority of cases to make mere assumption of its existence unnecessary, and moreover in those doubtful cases in which physical examination proves insufficient, the microscope will render valuable assistance.

The characteristics of the swelling itself, its relation to the substance of the breast, to the skin and to the thoracic wall must all be carefully investigated. If it be hard and firm in consistency, irregular in outline, diffuse instead of distinctly localized, fixed within the substance of the gland so that it is impossible to move the one without moving the other, then in all probability it is malignant. If in addition to these characteristics there be unnatural fixation of the subcutaneous tissues, fastening the skin over the indurated area and giving it a slightly thickened, roughened appearance, which a French surgeon has not inaptly compared to orange peel, evidence almost pathogomonic of malignancy is present. In examining a patient for this sign try to pick up the skin over the swelling; if adherent it cannot be raised. Also try to make the skin slip over the mass and try to move the mass under the skin; if adherent these manipulations will likewise be impossible. At a later stage in the evolution of the disease the breast becomes partly fixed to the pectoral fascia and after the latter has become invaded to the thoracic wall itself. To determine whether its mobility is reduced, place the flat of the hand upon the breast and try to move it in both directions laterally; then try the same manoeuvre with the patient's arm abducted, also try to move the gland up and down and lastly while her arm is in the same position steady her elbow with the other hand and have her try to bring her arm to her side. These manipulations will enable the examiner to detect beginning fixation and consequent diminished movability. Of course in later stages of the disease fixation is so apparent that the latter manipulations are not necessary, the breast being plastered, so to speak, to the underlying structures.

Retraction of the nipple, while an important sign if present, will be found only in those cases in which the tumor lies underneath the areola. If the growth begins in this situation or close to it, naturally the sign will manifest itself earlier than if it begins in another area of the gland. The most usual site is the upper outer quadrant, and as the neoplasm increases in size and extends downwards it not infrequently exerts sufficient traction upon the trabeculæ to draw the nipple slightly upwards and place it upon a higher level than the one on the opposite side. This sign, which should always be looked for in the early cases, precedes retraction of the nipple. About a year ago, Dr. Parker Syms, of New York, in discussing a paper on breast cancer called attention to this sign and stated that he had never seen it mentioned in print. It was, however, mentioned by Dr. Schwartz, of Paris, in a short article published in December, 1910, and there can be no doubt that many careful observers have noticed it in years past.

With reference to enlargement of the axillary lymph nodes, it may be stated that they are not sufficiently diseased in the early cases to be felt upon palpation through the skin and axillary fascia. If they can be distinctly palpated, then it is safe to conclude that the malady is already far advanced and that the patient's chances of long freedom from recurrence are slight. True it is that they are almost invariably found to be larger than normal at operation, although they are not big enough to be distinctly felt through the axillary tissues. Microscopic examination of a node apparently normal sometimes shows it to be already infiltrated with carcinoma. Thus it is seen that the absence of palpable nodes in no wise points to the benignity of an indurated mammary enlargement.

Pain is a matter of no consequence in beginning mammary carcinoma, although unfortunately the laity believe it to be an invariable accompaniment of all forms of malignant disease. Would that it were! As a matter of fact it is pretty safe to say that an indurated area in the breast which is painful is not malignant.

While the preponderance of cases occur in women past middle life, this fact must not make us forget that younger women are also subject to the disease. In five thousand cases which I collected and analyzed with reference to age incidence, twelve per cent. were in women between thirty and forty and nine per cent. in women between twenty and thirty. Just because a woman is young it must not be concluded that a tumor in her breast is benign. At a recent meeting of the New York Surgical Society, Dr. Ellsworth Eliot, Jr., asked the members to state their experience in this matter. Of those present there were five who had cases in women between the ages of twenty-one and twenty-eight. One gentleman said that Dr. Brewer had had a case in a child of eleven. That of course is unique.

Why speak of edema of the entire breast, of ulceration, of swelling of the arm, of enlarged supraclavicular lymph nodes, of cachexia, emaciation and the other signs of advanced malignancy? They are seen all too often and their significance is but too well known. They show that delay, dillydallying and procrastination have allowed the most opportune time for intervention to pass by; surely they spell the doom of the patient. Whose fault is it? The patient's? Yes, often times. The doctor's? In a certain percentage of cases, alas, the answer must be affirmative. The report of the Cancer Commission shows that cancer patients are held on an average for thirteen months before they are turned over to the surgeon. I for one, however, have too high an opinion of the great body of doctors at large ever to believe that they alone are culpable. Sometimes it is the fashionable consultant, the hospital physician, or even the professor of internal medicine who, when his opinion is sought by colleague and patient, doles out the advice, "Let us wait and see what will happen"—in other words, he means let us wait and see if the patient's breast will become fastened to her thoracic wall, if a mass will form in her axilla, if she will become pale and emaciated, develop internal metastases and die despite a possible belated effort to save her. Gentlemen, the hope of the future lies in better co-operation between patient, doctor and surgeon. Impress upon the patients the seriousness of their condition, get them to consent to operative treatment at once, and the results will be more and more satisfactory as time goes by. The progressive surgeons look to the general practitioners for help.

A few words about differential diagnosis. The condition with which early carcinoma is most likely to be confounded is chronic cystic mastitis, or abnormal involution, as it is now more frequently called. The nodules characterizing this condition are hard, irregular and intimately connected with the substance of the gland, yet their contour can be outlined. They are often painful and are sometimes tender under pressure. Occasionally they may develop simultaneously in different parts of the breast. Sometimes this condition is the sequel of acute inflammatory troubles, in which case its nature will be more easily recognized than when it develops irrespective of such precursors. Very often there is discharge from the nipple, or fluid can be squeezed out when the latter is pressed upon. In the more advanced cases it may be possible to detect small collections of fluid in the substance of the hardened areas. It is in this condition that microscopic diagnosis renders us the greatest service. The condition is distinctly a surgical one for the reason that it may become malignant. Greenough and Hartwell found carcinomatous degeneration in ten per cent. of the cases they examined. Others have found it in a higher percentage. Microscopic examination of a frozen section made by a skilled pathologist while the surgeon is operating will enable the latter to decide whether simple amputation of the breast will suffice or whether he need proceed with the complete and radical operation. Of course such an examination must not be intrusted to a mere tyro in laboratory methods. Even in the hands of the most expert it cannot be so thorough as the routine examination of many sections of fixed tissue, but still it is of sufficient certainty to be of great help. I am informed that this method has been used at the Mayo clinic with the most satisfactory results.

Diffuse syphilitic infiltration of the breast may also be mistaken for cancer, and no doubt breasts thus diseased have been removed under mistaken diagnosis. Fortunately it usually occurs during the late secondary period of infection, at a time when other manifestations are present. Were it not for this circumstance diagnosis would be even more difficult. Gummata resemble benign rather than malignant tumors and should not be mistaken for the latter. Still the mistake has been made.

The distinctive feature about tuberculosis is early involvement of the axillary glands, which are prone to break down before softening of the diseased areas in the breast takes place. In the discrete form of the disease, too, multiple areas of induration are present, a circumstance which precludes the possibility of the trouble being carcinomatous.

Finally, it must be borne in mind that there are some cases so obscure as to make clinical diagnosis impossible in the early stages. According to good authority the manifestatioins are so ill-defined in about ten per cent. of all cases that recourse must be had to the microscope to learn the exact nature of the trouble. That carcinomatous cysts, together with abnormal involution, constitute the bulk of such cases is not to be doubted.

Now as to treatment. It would be interesting to trace the evolution of the more complete operations, to show how they were improved step by step, and especially to point out how the percentages of cures increased *pari passu* with amplification of the operations; also to narrate the epoch-making work of Meyer and Halsted, and pay tribute again to the ingenuity of Warren. Time, however, does not permit these things, so a brief descriptioin of the procedure referred to in the beginning of this paper must suffice. During the years 1906 and 1907 it was my good fortune to be associated in the investigation of this subject with Professor William L. Rodman, who, beginning his work more than thirty years ago under the guidance of the late illustrious S. W. Gross, the pioneer in rational breast surgery in this country, received an inspiration which has never waned, and which, if I mistake not, has finally resulted in the perfectioin of an operative technique that leaves little to be desired from the standpoints of anatomy, pathology and surgery. It is the product of profound thought and hard labor. It was worked out on the cadaver before it was practised upon the living, and it is now done a little differently from the way it was first performed.

The first incision starts about an inch below the clavicle and two fingers' breadth internal to the sulcus between the deltoid and the great pectoral muscles and is carried down well onto the margin of the anterior axillary fold. It divides all the superficial tissues and exposes the anterior surface of the pectoralis major. The index finger of the left hand is carried beneath the muscle and made to push its way out between the clavicular and thoracic portions, after which the muscle is divided close to its insertion. Thus the clavicular portion is left, you will see. The dissection is then continued deeper and the pectoralis minor is shortly exposed, being in turn divided close to its insertion into the coracoid process. I may say now that the man who can make a perfect dissection of the axilla with the pectoralis minor in situ has yet to be born. It cannot be well done on the cadaver, much less on the living. The costocoracoid membrane, which fills in the space between the subclavius and the pectoralis minor, lies over the apex of the axilla and it, too, should be divided, whereupon the whole space from apex to base will be laid wide open. It is then cleaned of its contents,—fat, fascia and glands—the dissection being begun above and carried downwards. A piece of gauze wound around the finger forms the best instrument for this dissection, after the fascia has once been divided with a sharp knife external to the axillary vessels and brachial plexus. The branches of the axillary artery and their accompanying veins are secured and divided as they are brought into view. The axillary dissection completed, another incision, elliptical in outline, is begun at the middle of the first one and is carried around the breast from before backwards going right down through both pectorals to the serratus magnus. The lower part of this cut is extended down onto the abdomen, so that the anterior layer of the sheath of the rectus may be divided transversely. I carry it even lower down than does Rodman himself and excise a piece of the fascia. This division or removal of the deep fascia is made to destroy the integrity of the subfascial lymphatic plexus through which, according to Mr. Handley, of London, direct extension of carcinoma takes place. Accepting Mr. Handley's views on this matter, I sometimes remove the lower part of the serratus magnus itself in addition to a piece of the fascia. In the second incision the skin is widely undermined. The wound made by these two incisions is very extensive, and anyone seeing it for the first time is bound to think that there will be difficulty in closing it. As a matter of fact such is not the case. The straight cut is closed first, then the elliptical one. I have done a secondary operation on a patient primarily operated upon in a late stage of her disease and still closed the wound. It was necessary, however, to make the relaxation incisions of Warren.

The advantages of this operation may be summarized as follows: it attacks the axilla first, thus making possible the removal of diseased tissue from without inwards; it exposes the axilla better than any other method; it does not encroach upon the arm and consequently does not leave a scar which interferes with the natural movements of that limb; it lessens hemorrhage by permitting ligation of the axillary vessels close to their origin during the early stages of the operation; it saves times; it permits extensive removal of tissue without necessitating skin grafting. These are points in which it excels.

I will here take occasion to state that the first or straight incision used in this operation affords the best route of attack upon axillary tumors. I have used it twice for this purpose and once for the removal of a malignant growth of the pectoral muscle invading the axilla. I believe I was the first one to employ it for this purpose, and as it has proved so eminently satisfactory, I desire to call attention to it.

Of course no one can say when a cancer patient is cured. The three year limit is entirely too short, and likewise the disease may return long after five years. A patient who remains well for three years, however, has good expectations for the future. The possibility of local or internal recurrence in all cases, even those operated upon early, must be recognized. Neoplasms which have grown rapidly are more likely to return than those which have developed slowly. At all events the best results are to be expected when the patient is operated upon early in the course of the disease. Give us patients with movable breasts and we will give you a greater number of cures.

1117 Spruce Street.

